# Comparative Assessment of Crohn's Disease Activity Using Magnetic Resonance Enterography and Endoscopy

**DOI:** 10.7759/cureus.61247

**Published:** 2024-05-28

**Authors:** Waleed Alkhaldi, Mohamed Sherif Elsharkawy, Ali H Bashaib, Hussein Alsakkaf, Meshari A Alali, Bandar Rashed Alfheed, Bader A Alahaideb, Mohammed M Alharbi, Saud M Alzahrani

**Affiliations:** 1 Medicine, Majmaah University, Al Majma'ah, SAU; 2 Radiology and Medical Imaging, King Saud University Medical City, Riyadh, SAU; 3 Gastroenterology and Hepatology, Prince Mohammad Bin Abdulaziz Hospital, Riyadh, SAU; 4 Medical Imaging, King Fahd Specialist Hospital, Buraydah, Buraydah, SAU; 5 Medical Imaging, King Saud University Medical City, Riyadh, SAU; 6 Medicine, Umm Al-Qura University, Al Qunfudhah, SAU; 7 General Physician, Prince Meshari Bin Saud General Baljarshi Hospital, Al Bahah, SAU

**Keywords:** diagnostics, endoscopy, crohn’s disease, enterography, mri

## Abstract

Introduction

Magnetic resonance enterography (MRE) has emerged as a promising technique for evaluating the extent and severity of Crohn's disease activity. To compare how we measure Crohn's disease activity with MRE and endoscopy.

Material and methods

We retrospectively reviewed MRE studies of 60 patients with suspicious Crohn's disease who underwent 1.5-T MRI examinations (T1-weighted images pre- and post-IV contrast medium administration and T2-weighted images) and endoscopy within one month, and they were evaluated by one radiology consultant with experience of 17 years. Endoscopy was used as the reference standard for diagnosing active Crohn's disease cases. Data analysis was performed using the websites (www.graphpad.com and www.medcalc.org) and Microsoft Excel (Microsoft® Corp., Redmond, USA).

Results

A total of 35 patients were included in the study. The remaining 25 patients were excluded either due to non-available data in the endoscopy report or cases of non-Crohn's disease. The MRI examinations were reviewed by one radiology consultant and revealed 27 active and eight non-active Crohn's disease cases compared to 30 active and five non-active Crohn's disease cases in endoscopy. The sensitivity of MRI in detecting active cases of Crohn's disease compared to endoscopy was 83.3% and the specificity of 60%. The strength of agreement between both methods was fair to good (Kappa = 0.347, p-value = 0.4497, Chi-squared = 0.571 with one degree of freedom).

Conclusion

MRE statistically has a good impact on the assessment of Crohn's disease as well as endoscopy with the parameters used in this study. Non-invasiveness and the changes of activity seen in the bowel proximal to the ileocecal junction undetectable by endoscopy make MRE more practically applicable in this aspect.

## Introduction

Crohn's disease, a chronic inflammatory condition of the gastrointestinal tract, presents a complex diagnostic challenge due to its varied clinical manifestations and the need for accurate localization and assessment of disease activity [1-3]. In recent years, medical imaging has played a pivotal role in enhancing our understanding of Crohn's disease, enabling more precise and non-invasive methods for diagnosis and monitoring [4,5]. Among these imaging modalities, magnetic resonance enterography (MRE) has emerged as a promising technique for evaluating the extent and severity of Crohn's disease activity [6].

This study investigates the efficacy of MRE as an assessment tool for Crohn's disease in comparison to the conventional endoscopic approach. While endoscopy remains a cornerstone in the diagnosis and surveillance of Crohn's disease, its invasive nature and limitations in assessing extraluminal manifestations necessitate exploring alternative imaging methods [7,8]. MRE, a non-invasive and radiation-free imaging technique, provides detailed visualization of the small bowel and surrounding structures, offering a comprehensive evaluation of disease involvement. The objective of this research is to systematically compare the diagnostic accuracy, sensitivity, and specificity of MRE with traditional endoscopy in detecting and characterizing Crohn's disease activity. By examining the concordance between these two modalities, we seek to elucidate whether MRE can serve as a reliable alternative or complementary tool for the assessment of Crohn's disease, potentially revolutionizing the diagnostic landscape and improving patient outcomes [9-11].

Through a comprehensive analysis of relevant literature, clinical cases, and imaging data, this study endeavors to contribute valuable insights that can inform clinical decision-making and guide the implementation of more patient-friendly diagnostic strategies for Crohn's disease [12]. As we delve into the comparative assessment of MRE and endoscopy, we anticipate shedding light on the strengths and limitations of each modality, ultimately paving the way for more personalized and effective management of individuals affected by this challenging inflammatory condition. This study aims to compare the activity of Crohn’s disease measured using MRE with that of endoscopy as the reference standard.

## Materials and methods

We conducted a retrospective review of MRE studies involving 60 patients with suspected Crohn's disease, all of whom underwent 1.5-T MRI examinations. The study was approved by the Deanship of Scientific Research at Majmaah University, Al Majma'ah, Saudi Arabia with IRB no. MUREC-June 8/COM-2023/21-6. The MRI protocol included the acquisition of T1-weighted images both pre- and post-intravenous contrast medium administration, as well as T2-weighted images to provide a comprehensive assessment of the small bowel and surrounding structures. All imaging studies were conducted within a one-month timeframe of the corresponding endoscopic procedures. The interpretation of the MRE studies was carried out by a single radiology consultant with extensive experience, boasting a 17-year track record in gastrointestinal imaging. The active and inactive disease on MRI was classified according to the wall thickening, mural enhancement, and submucosal edema; whereas the active and inactive disease on endoscopy was classified on Crohn's Disease Endoscopic Index of Severity (CDEIS) and Simple Endoscopic Score for Crohn's Disease (SES-CD).

To establish a robust reference standard for diagnosing active Crohn's disease cases, conventional endoscopy was employed. Endoscopic evaluations were performed following established clinical protocols, and the findings were meticulously recorded. The use of endoscopy as the reference standard ensured a thorough and clinically relevant assessment of Crohn's disease activity, considering its status as the conventional gold standard for mucosal evaluation in the gastrointestinal tract. The features of disease activity in MRE include wall thickening, mural enhancement, and submucosal edema. Features of disease activity in endoscopy include worsening of ulcers either increase in number or size or distribution the presence of stricture/stenosis or deformity. The diagnosis of Crohn’s disease was made based on clinical manifestations (including chronic abdominal pain with chronic diarrhea, weight loss, or symptoms suggestive of complications of the disease like bowel obstruction or an abdominal collection, bowel perforation, or malabsorption) and endoscopic findings, (terminal ileum aphthous ulcer, skip lesion, stenosis, and deformity of the ileocecal valve) and histopathological findings (chronic cryptitis, crypt distortion, granuloma) and high inflammatory markers (high ESR, high CRP).

In a research conducted by Hafeez et al., it was found that 80% of participants exhibited active small bowel disease. Utilizing this percentage in the formula Z = 1.96×1.96×0.80×0.20/(0.01)×(0.01), the calculated figure amounted to 31 [12]. However, accounting for a 10% attrition rate, the approximation was adjusted to approximately 35.

Data analysis was conducted using established statistical tools and platforms, specifically the websites www.graphpad.com and www.medcalc.org. These platforms were chosen for their reliability and versatility in performing statistical analyses pertinent to medical research. Additionally, Microsoft Excel (Microsoft® Corp., Redmond, USA) was employed for data management and organization, facilitating the compilation and systematic analysis of the data gathered from both the MRE studies and the endoscopic evaluations. The combined use of these tools aimed to ensure a comprehensive and rigorous statistical analysis, providing meaningful insights into the comparative efficacy of MRE and endoscopy in diagnosing and assessing the activity of Crohn's disease. The level of significance was kept at 5%. The demographic details and findings of the MRI were presented using descriptive statistics. The agreement between MRI and endoscopy was tested using Kappa statistics. The diagnostic accuracy of MRI was analyzed using the receiver operating characteristic (ROC) curve method.

## Results

A total of 35 patients were included in the study. The remaining 25 patients were excluded either due to non-available data in the endoscopy report or cases of non-Crohn's disease. MRI examinations were reviewed by one radiology consultant and revealed 27 active and eight non-active Crohn's disease cases compared to 30 active and five non-active Crohn's disease cases in endoscopy. The mean age of the study participants was 27.4±9.5 years. There were 17 (48.6%) of females and 18 (51.4%) of males.

**Figure 1:**
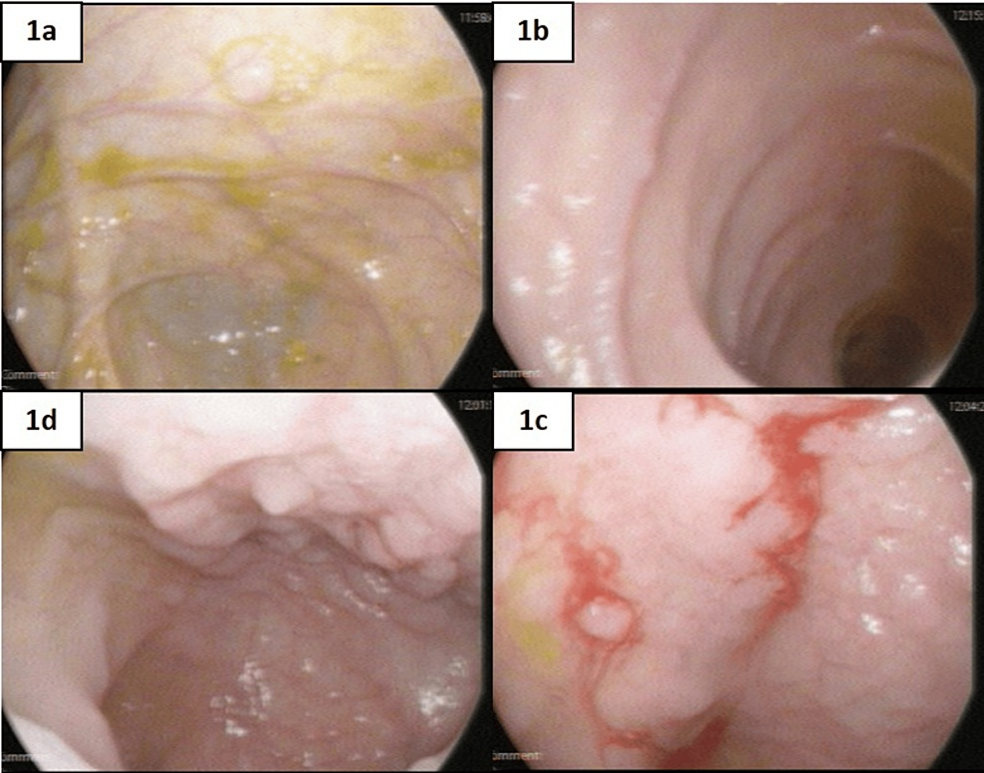
Endoscopic analysis Figures 1a-1b represent the normal colonic mucosa and figures 1c-1d represent the terminal ilium aphthous ulcers

**Figure 2:**
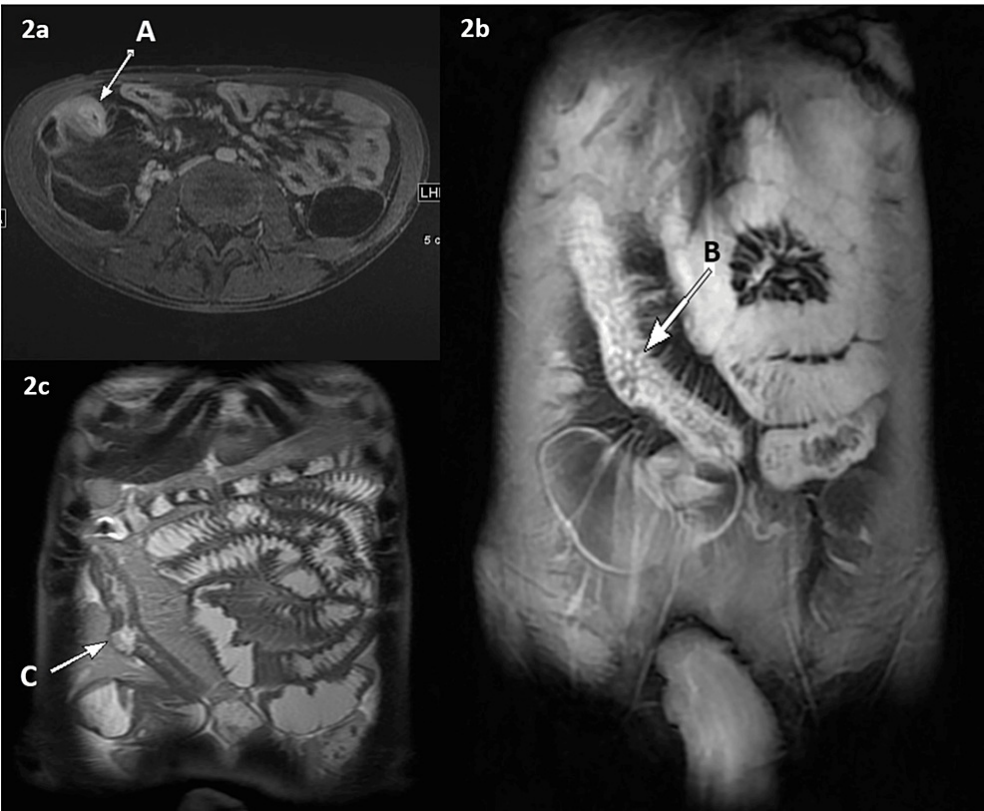
MRI image analysis Axial (2a) and coronal (2b) T1 fat-suppressed post-contrast images of the abdomen and pelvis and coronal (2c) T2 with oral volume demonstrate long segment of circumferential wall thickening and mucosal enhancement involving the distal ileum A: an entero-enteric fistula seen in the right iliac fossa; B: mucosal enhancement and congested mesenteric vessels; C: at least one deep mucosal ulceration seen in the distal ileum

Figure 1 and Figure 2 represent the endoscopic and MRI images of one of the study subjects evaluated. Figure 2 shows the luminal narrowing and dilated small bowel loops measuring up to 3.5 cm. There is mesenteric vascular congestion as well.

The MRI findings from our study cohort reveal a spectrum of manifestations in Crohn's disease patients. These include suboptimal features, deep ulcers, and abscesses. Additionally, strictures, edema, and vascular enhancement were observed. Mucosal and submucosal enhancement, fibrosis length, and fistulae were also prevalent. Some patients exhibited collections on MRI, while many had lymph nodes, with varying sizes. Anatomically, manifestations were commonly found in the terminal ileum, other small intestine sites, and the colon. Severity was noted in some cases, with thickness variation across subjects. Crohn's disease classification included features consistent with inflammatory, fibrosing, and fistulizing types. This analysis provides valuable insights into the diverse phenotypic presentations of Crohn's disease within our study population (Table 1).

**Table 1:** MRI findings showing the diverse phenotypic presentations of Crohn's disease

MRI		Frequency	Percent
Suboptimal	No	32	91.4
	Yes	3	8.6
Deep ulcer	No	34	97.1
	Yes	1	2.9
Abscess	No	28	80.0
	Yes	7	20.0
Stricture	No	29	82.9
	Yes	6	17.1
Edema	No	24	68.6
	Yes	11	31.4
Enhancement	Vascular	16	45.7
	Mucosa	11	31.4
	Sub-mucosal	6	17.1
	Lymph nodes	2	5.7
Fibrosis length	No	32	91.4
	Yes	3	8.6
Fistula	No	23	65.7
	Yes	12	34.3
Fistula type	Enterocolic/enteroenteric	3	8.6
	Enterocutaneous	4	11.4
	Enterovesicular	4	11.4
	NA	24	68.6
Collections	No	33	94.3
	Yes	2	5.7
Lymph node	No	14	40.0
	Yes	21	60.0
Lymph node size	<1 cm	24	68.6
	>1 cm	11	31.4
Site	Terminal ileum	28	80.0
	Small intestine	11	31.4
	Colon	5	14.3
Severity	No	34	97.1
	Yes	1	2.9
Thickness	1-3 mm	11	31.4
	>3-5 mm	14	40.0
	>5-7 mm	6	17.1
	>7 mm	4	11.4
Type of Crohn’s disease	Inflammatory	11	31.4
	Fibrosing	6	17.1
	Fistulizing	8	22.9
	Not applicable	10	28.6

The MRI observations revealed a range of mural T2 characteristics, with bowel segments exhibiting dark, grey, or lighter tones. The perimural T2 characteristics varied, with cases showing darker or brighter signals, and occasionally a fluid rim. The contrast-enhanced patterns displayed homogenous appearances, mucosal contrast, or layered patterns. The degree analysis revealed a normal or decreased contrast, particularly near vessels. Lymph node measurements indicated varying sizes, with contrast-enhanced characteristics typically near vessels. Approximately half of the cases displayed combined wall thickening, with an average thickness reported. Crohn's Disease Activity Score (CDAS) averaged a moderate level, with enhancement, edema, and ulcers commonly observed. These MRI findings offer detailed insights into mucosal and perimural characteristics, contrast patterns, and associated parameters, contributing to a comprehensive assessment of Crohn's disease in the study cohort (Table 2).

**Table 2:** MRI findings showing the mucosal and perimural characteristics, contrast patterns, and associated parameters contributing to the comprehensive assessment of Crohn's disease in the study cohort LN C+: contrast-enhanced lymph node

MRI		Frequency	Percent
Mural T2	Dark	17	48.6
	Grey	14	40.0
	Light	4	11.4
Perimural T2 signal	Dark	24	68.6
	Bright	10	28.6
	Fluid rim <2 mm	1	2.9
C+ pattern	Not applicable	7	20.0
	Homogeneous	16	45.7
	Mucosal	7	20.0
	Layered	5	14.3
C+ degree	Normal	8	22.9
	<< vessel	23	65.7
	< vessel	4	11.4
Lymph node measurement	No	15	42.9
	<1 cm	11	31.4
	>1 cm	9	25.7
LN C+	< vessel	34	97.1
	> vessel	1	2.9
Combined wall thickening	No	18	51.4
	Yes	17	48.6
Wall thickening (mm)		8.8±1.6	
Crohn's Disease Activity Score		3.8±1.7	
Enhancement		21	60.0
Edema		20	57.1
Ulcers		18	51.4

The MRI correctly identified 25 active cases out of a total of 30 active cases and three non-active cases out of a total of five non-active cases of Crohn's disease (Table 3). The agreement between MRI and endoscopy in the diagnosis of Crohn's disease was fair (k = 0.347). 

**Table 3:** Strength of agreement between MRI and endoscopy findings *p<0.05 indicates a significant agreement

		Endoscopy		Total	Kappa, p-value
		Active	Non-active		
MRI	Active	25	2	27	0.347, 0.033*
	Non-active	5	3	8	
Total		30	5	35	

The sensitivity of MRI in detecting active cases of Crohn's disease compared to endoscopy was 83.3% and the specificity of 60% (Table 4). There was a high probability (positive predictive value (PPV): 93%) that the patient had active Crohn's disease. The accuracy of MRI in detecting both positive and negative cases of Crohn's disease compared to endoscopy was 80%. 

**Table 4:** Sensitivity analysis of MRI in comparison to endoscopy PPV: positive predictive value; NPV: negative predictive value

Objective measures	%
Sensitivity	83%
Specificity	60%
PPV	93%
NPV	38%
Accuracy	80%

**Figure 3:**
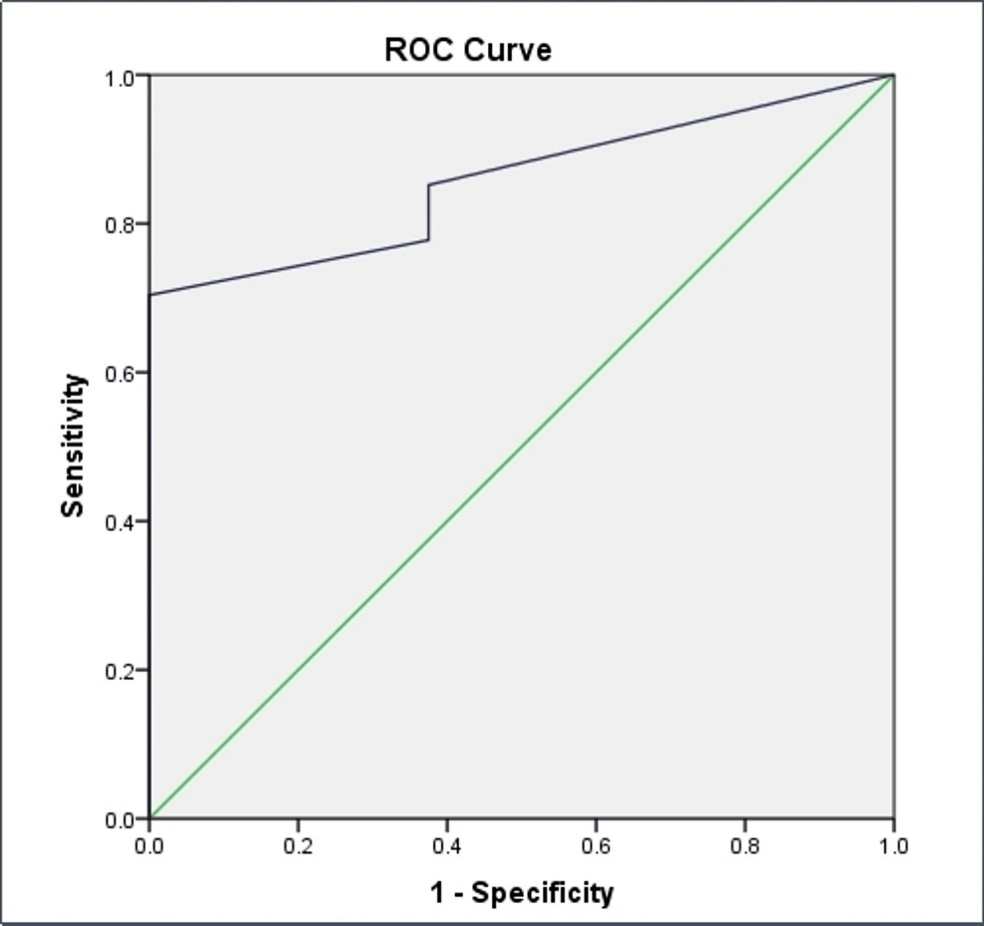
Receiver operating characteristic (ROC) analysis

With an area under the curve (AUC) of 0.856, this suggests that MRI is a reliable diagnostic tool for detecting cases of Crohn's disease, with a high probability (approximately 85.6%) of correctly identifying affected individuals (Table 5, Figure 3).

**Table 5:** Receiver operating characteristic (ROC) analysis CDAS: Crohn's Disease Activity Score

Area under the curve	95% CI	p-value	CDAS cut-off value
0.856	(0.73-0.98)	0.002	3.6

## Discussion

The present study aimed to compare the diagnostic performance of MRE and endoscopy in assessing Crohn's disease activity. Of the initially identified 60 patients, data from 35 patients were included in the final analysis due to non-availability of data in the endoscopy reports or instances of non-Crohn's disease cases among the exclusions. The mean age of the study participants was 27.4±9.5 years, with a nearly equal distribution of 48.6% females and 51.4% males, ensuring a balanced representation of gender in the cohort.

MRI examinations, conducted and evaluated by a single experienced radiology consultant, demonstrated 27 cases with active Crohn's disease and eight cases classified as non-active, compared to 30 active and five non-active cases identified through endoscopy. This discrepancy in case classification prompts further exploration of the strengths and limitations of each modality.

The comprehensive analysis of MRI findings revealed a diverse range of Crohn's disease manifestations within the study cohort. These included deep ulcers, abscesses, strictures, edema, vascular engorgement, mucosal and submucosal enhancements, evaluation of fibrotic changes, and various types of fistulae. Noteworthy was the detailed anatomical distribution of the disease, with a predominant occurrence in the terminal ileum. The differentiation based on severity, thickness, and Crohn's disease classification provided a nuanced understanding of the phenotypic presentations within the study population.

The MRI observations complemented the endoscopic findings by providing valuable insights into mucosal and perimural characteristics, contrast patterns, and additional parameters contributing to the comprehensive assessment of Crohn's disease. The identified features included mural T2 characteristics, perimural T2 characteristics, contrast-enhanced patterns, lymph node measurements, wall thickening, and the prevalence of ulcers.

The comparison between MRI and endoscopy yielded fair to good agreement, as indicated by a Kappa value of 0.347. The sensitivity of MRI in detecting active Crohn's disease was noteworthy at 83.3%, while specificity stood at 60%. The ROC analysis, reflected in the AUC value of 0.856, suggested a robust discriminatory ability of MRI in distinguishing active cases of Crohn's disease.

In the investigation conducted by Khater et al., MRE exhibited a sensitivity of 82% and specificity of 80%, with PPV and negative predictive value (NPV) of 83% and 80%, respectively, in comparison to colonoscopy [13]. In contrast, colonoscopy demonstrated a sensitivity of 84% and specificity of 85%, with a higher PPV of 90% and an impressive NPV of 98% [14]. The MRE's capacity to detect subtle mucosal findings and abnormal enhancement was attributed to the contrasting signal intensities between luminal contents and the bowel wall. This capability aligned well with findings from studies by Casciani et al., Tillack et al., and Albert et al., suggesting that MRE yielded comparable results to traditional endoscopic techniques [15-17].

However, Khater et al. noted a limitation in their study related to the suboptimal depiction of inflammatory changes in the proximal small bowel [13]. Reduced patient compliance in ingesting the required amount of fluid hindered proper assessment of the proximal bowel. When bowel loops were adequately distended, sensitivities for wall thickness and wall enhancement reached 78% and 82%, respectively. These findings were in line with results reported by Sinha et al., who demonstrated sensitivities and specificities ranging from 83% to 91% and 86% to 100%, respectively [18]. Specific indicators such as Coomb’s sign, mesenteric adenopathy, and extra-intestinal complications exhibited high values of 92%, 89%, and 100%, respectively.

The study also highlighted MRE's high sensitivity in detecting inflammatory changes in the ileum, particularly the terminal ileum, with sensitivities reaching 92% and 95%, respectively. These results were consistent with findings from Yuksel et al., which demonstrated a sensitivity of 92% for assessing ileal Crohn’s disease [19]. The additional use of diffusion-weighted imaging (DWI) axial sequences, with b values at 0, 400, and 800, proved valuable in documenting acute inflammatory changes. Furthermore, the study revealed the effectiveness of MRE in demonstrating Crohn's disease complications, including transmural ulceration progressing to fistulae formation, with sensitivities between 83% and 84% and a specificity of 100%, aligning with results reported by Rieber et al. [20].

From our 30 cases, we found three perianal fistulas, two anterior wall fistulae, three enteroenteric fistulae, two enterovesical fistulae, and one enterocolic fistula. The exact size and location of perianal fistulae were seen on MRI, apart from one perianal fistula which was missed by MRE but clinically suspected and diagnosed on examination under anesthesia. We found that a dedicated high-resolution perianal MR protocol study is much superior in the detection of minute subtle tracts [21,22].

This study provides a comprehensive assessment of Crohn's disease using both MRE and endoscopy, offering valuable insights into the strengths and limitations of each modality. The observed agreement and diagnostic performance metrics, especially the high sensitivity and specificity of MRI, underscore its potential as a reliable alternative or complementary tool in the evaluation of Crohn's disease activity [23,24]. 

Though MRE can serve as a promising tool, it has certain inherent limitations when compared to endoscopy. The interpretation of MRE images can be subjective and may vary depending on the experience and expertise of the radiologist. Endoscopy, on the other hand, allows for real-time assessment by the endoscopist, potentially reducing interpretation variability. MRE may be more costly and less widely available compared to endoscopy, and it cannot obtain tissue samples for histological analysis, which is crucial for confirming the diagnosis of Crohn's disease and assessing disease activity. Further research and larger-scale studies are warranted to validate these findings and refine the integration of imaging modalities in clinical practice for enhanced diagnostic accuracy and patient care.

## Conclusions

MRE statistically has a good impact on the assessment of Crohn's disease as well as endoscopy with the parameters used in this study. Non-invasiveness and the changes of activity seen in the bowel proximal to the ileocecal junction undetectable by endoscopy make MRE more practically applicable in this aspect. MRE offers non-invasive assessment of the small bowel, and visualization of extraluminal complications, and can be particularly useful in cases where endoscopy is contraindicated or inaccessible. It provides complementary information to endoscopy and is valuable for assessing disease extent, detecting complications such as strictures and fistulas, and monitoring treatment response. Ultimately, the choice between MRE and endoscopy depends on various factors including clinical presentation, disease severity, accessibility, and patient preference. In many cases, both modalities may be used in conjunction to provide a comprehensive evaluation of Crohn's disease.
